# Characterization of the internal IRES element of the zebrafish connexin55.5 reveals functional implication of the polypyrimidine tract binding protein

**DOI:** 10.1186/1471-2199-9-92

**Published:** 2008-10-23

**Authors:** Mahboob Ul-Hussain, Rolf Dermietzel, Georg Zoidl

**Affiliations:** 1 Department of Neuroanatomy and Molecular Brain Research, Ruhr-University Bochum, Bochum, Germany; 2 Department of Cytology, Ruhr-University Bochum, Bochum, Germany; 3 International Graduate School of Neuroscience (IGSN), Ruhr-University Bochum, Bochum, Germany

## Abstract

**Background:**

Connexin55.5 (Cx55.5) is a gap junction protein with horizontal cell-restricted expression in zebrafish accumulating at dendritic sites within the receptor-horizontal cell complex in form of hemichannels where light-dependent plasticity occurs. This connexin is the first example of a gap junction protein processed to form two protein isoforms from a monocistronic message by an IRES mediated process. The nuclear occurrence of a carboxy-terminal fragment of this protein provides evidence that this gap junction protein may participate in a putative cytoplasmic to nuclear signal transfer.

**Results:**

We characterized the IRES element of Cx55.5 in terms of sequence elements necessary for its activity and protein factor(s), which may play a role for its function. Two stretches of polypyrimidine tracts designated PPT1 and PPT2 which influence the IRES activity of this neuronal gap junction protein were identified. Selective deletion of PPT1 results in an appreciable decrease of the IRES activity, while the deletion of PPT2 results in a complete loss. RNA-EMSA and UV-cross linking experiments showed that protein complexes bind to this IRES element, of which the polypyrimidine tract binding protein (PTB) was identified as one of the interacting partners with influence on IRES activity. These results indicate that PTB conveys a role in the regulation of the IRES activity of Cx55.5.

**Conclusion:**

Our findings indicate that the activity of the IRES element of the neuronal gap junction protein Cx55.5 is subject of regulation through flanking polypyrimidine tracts, and that the non-canonical trans-activation factor PTB plays an essential role in this process. This observation is of considerable importance and may provide initial insight into molecular-functional relationships of electrical coupling in horizontal cells.

## Background

Cap-dependent translation is not the only means by which mRNA translation can be initiated. The discovery of internal ribosome entry sites (IRES) in picornaviruses mRNA revealed that the small ribosomal subunit could bind within the mRNA in a cap-independent manner [1,2]. Because of this property, IRES elements provide an exception of the general mechanism of scanning from the 5' end of the cap structure to initiate eukaryotic translation. Multiple IRESs have subsequently been found on different viral mRNAs, and more recently in cellular mRNAs [3-10]. The presence of IRES elements in viruses provides them with the advantage to hijack the translational machinery of the host cell to favor the expression of foreign transcripts. Most of the cellular IRESs have been shown to function preferentially when cap-dependent translation is physiologically impaired. Consistent with this concept, IRES elements were active during γ-radiations [11], hypoxia [12] or amino acid starvation [13]. This led to the current hypothesis that IRES-mediated translation of certain mRNAs represents a regulatory mechanism that helps the cell to cope with transient stress.

Connexins form gap junctions, which are believed to convey a broad spectrum of functions, including the regulation of cell growth, cell differentiation and maintenance of tissue homeostasis [14]. Translational initiation of connexin genes has been regarded mainly in a cap-dependent manner, but recent reports have shown that connexins possess IRES elements [15], where in one case a single point mutation in an IRES element of the 5' untranslated region has been linked to Charcot-Marie-Tooth disease [16]. A recent report on connexin43 has shown that this gene use alternate splicing mechanism, which yields transcripts with different 5'-UTRs displaying different translational efficiencies [17]. Moreover, we reported on the presence of a unique internal IRES element in the coding region of the horizontal cell specific zebrafish Cx55.5, which results in an internal translation of a carboxy-terminal domain (p11-CT) [18,19]. The presence of such IRES elements in the coding region of eukaryotic genes has only recently become apparent [20-22].

Regulation of a typical caped eukaryotic mRNA by modulating the activity of critical translational initiation factors, eIF4E and eIF4F, is well known [23]. However, the translational regulation of the IRES containing mRNA is still less understood. To get insight into the regulation of the IRES elements in cellular genes, it becomes imperative to identify the motifs within the IRES's scaffold, which are important for its function. In addition to the requirement of the canonical initiation factors, the requirements of some non-canonical trans-acting factors have been found important for the function of IRES elements [24-26].

In the present study, we extent a previous report on the presence of an internal IRES element in the coding region of Cx55.5 by characterizing the IRES site in terms of sequence elements important for IRES activity and putative trans-acting factors that could modulate the IRES function. Our findings indicate that the activity of the IRES element is subject of regulation through flanking polypyrimidine tracts, and that PTB seems to be an essential RNA binding factor involved in this process. This observation is of considerable importance since it adds a further facet to the widespread function of PTB. On one hand PTB has been characterized as a crucial factor involved in the non-canonical trans-activity mechanisms. There, PTB was initially discovered as a splicing factor due to its ability to bind to polypyrimidine tracts at 3' splice sites [27-30]. Here, our finding supports the previously described role of PTB in IRES mediated translation [26,31-33], which in our case consists of an internal segment that allows alternate translation of a carboxy-terminal isoform with the capability to translocate to the nucleus. This finding may provide initial insight on molecular-functional relationships of electrical coupling in horizontal cells, which show a unique light dependent plasticity. Changes of the interneuronal coupling mediated by electrical synapse proteins in response to light adaptation, receptive field shaping and structural plasticity are known as a paramount feature of the outer retina [34,35]. The regulation of these processes is not understood at the molecular level but information transfer to the nucleus by a locally generated messenger deriving from an electrical synapse protein may comprise a feasible molecular determinant in a yet uncharacterized signalling pathway. On the basis of our results it is tempting to speculate whether the Cx55.5 CT domain shows a light stimulus dependent translocation and exert its function by interaction with DNA, RNA and/or other nuclear proteins thus modulating gene regulation making plasticity of horizontal cells possible.

## Results

### Cx55.5 internal IRES element activity is modulated by two polypyrimidine tracts

**Figure 1:**
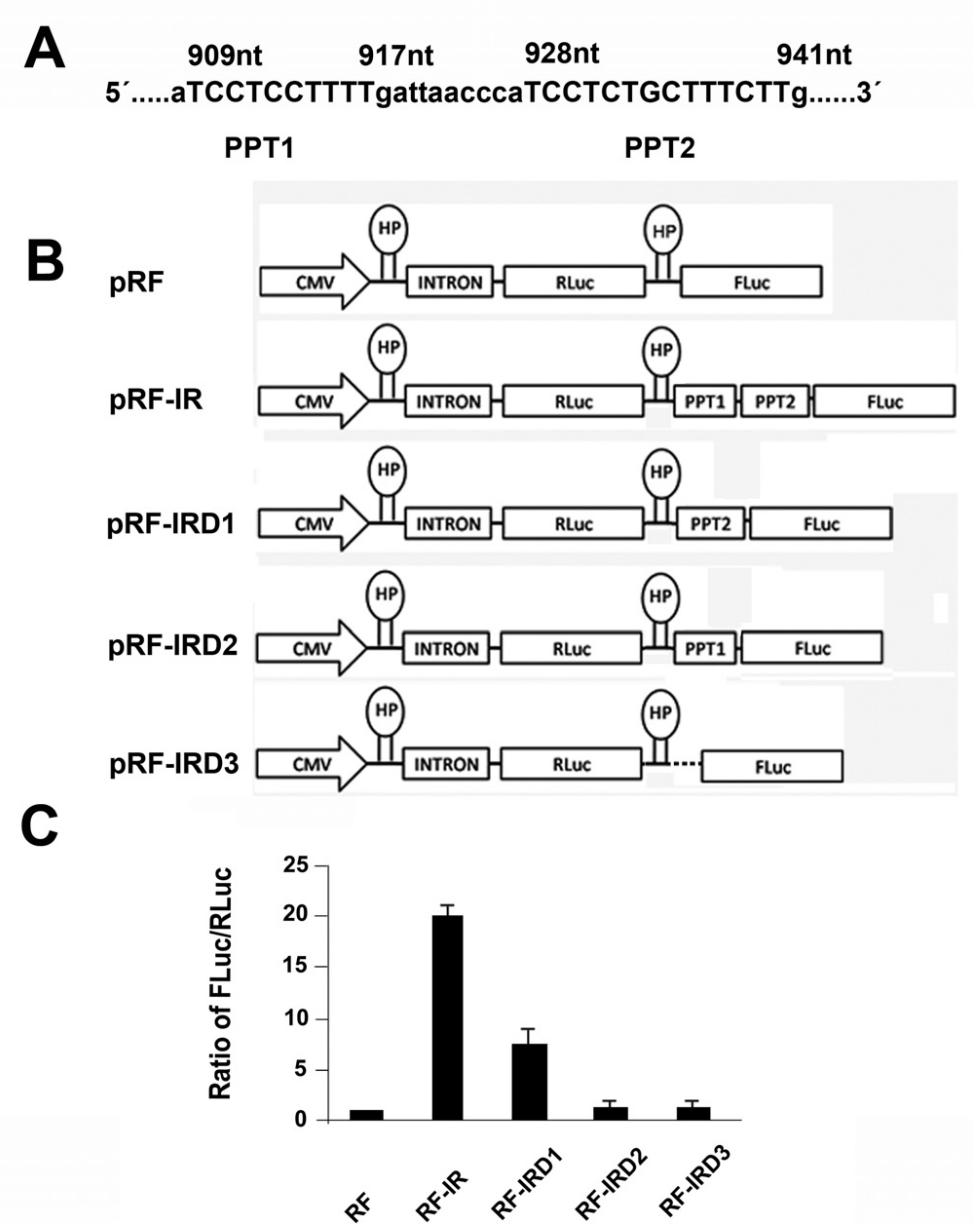
**Deletion of polypyrimidine tract elements reveal a significant reduction in IRES mediated activitys of Cx55.5**. A) Partial DNA sequence of the Cx55.5 IRES element with polypyrimidine tract 1 (PPT1) and polypyrimidine tract 2 (PPT2) (underlined by bars) is depicted. The relative position of each element relative to the start nucleotide of the Cx55.5 coding region is shown. B) Schematic representation of the Di-cis constructs applied: pRF, control vector having *Rennila *luciferase as first cistron (RLuc) and *Firefly *luciferase as downstream cistron (FLuc) with the first cistron under the control of the CMV promoter activity. pRF-IR; wild type IRES containing Di-cis construct, with the IRES element placed between first and second cistron. pRF-IRDel1 = PPT1 deleted, pRF-IRDel2 = PPT2 deleted, pRF-IRDel3 = PPT1, PPT2 and the intervening sequence deleted. HP = hairpin structure C) IRES activity of the constructs in transiently transfected N2A cells. IRES activity is represented as the ratio of *Firefly *to *Rennila *luciferase activities (FLuc/RLuc) with the activity of control vector set to "1". Each construct was tested 4 times and each experiment was done in triplicates. Data are represented as mean of ± SEM.

**Figure 2:**
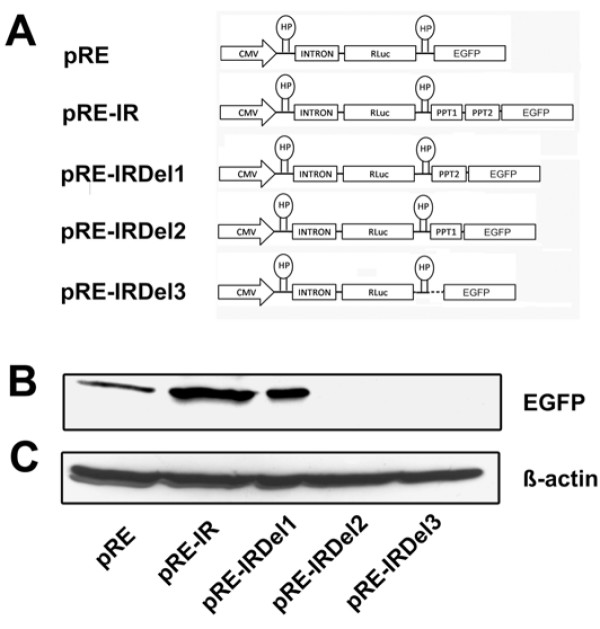
**Western blot of wild type IRES element and its deletion mutants substantiate the efficiency of the PTB elements**: A) Schematic representation of Di-cis constructs used for the Western blot analysis. pRE; control vector having RLuc as first cistron and EGFP as downstream cistron, pRE-IR; wild type IRES containing Di-cis construct, pRE-IRDel1; PPT1 deleted, pRE-IRDel2; PPT2 deleted and pRE-IRDel3; PPT1 and PPT2 deleted IRES construct. B) Western blot of the constructs transiently transfected into N2A cells. 30 μg of cytosolic total proteins were resolved by 10% SDS-PAGE. Immunodetection was done using anti-GFP (1:2000) as primary antibody and peroxidase labeled anti-mouse IgG (1:7,500) as secondary antibody. C) The detection of β-actin served as internal loading control.

We reported recently that the carboxy-terminal domain (p11-CT) of the zebrafish Cx55.5 can be internally translated from an IRES element present in the coding region of the full length Cx55.5 mRNA [19]. A detailed sequence analysis of this element revealed the presence of two stretches of polypyrimidine tracts named polypyrimidine tract 1 (PPT1, nt 909–917) and polypyrimidine tract 2 (PPT2, nt 928–941) (Fig 1A). We hypothesized that both sequence elements could contribute to IRES activity and generated plasmid constructs with deletions of PPT1 or PPT2 separately or in combination with the IRES element (IR) containing the Di-cistronic vector pRF-IR. The control Di-cis vector pRF along with wild type IRES Di-cis vector pRF-IR and the various deletion mutant constructs of the IRES element, pRF-IRDel1, pRF-IRDel2 and pRF-IRDel3 (Fig. 1B), were transiently transfected into N2A cells and the *Rennila *and *Firefly *luciferase activity measured 48 hrs post transfection. The IRES activity was calculated as the ratio of *Firefly *luciferase to *Rennila *luciferase activity (FLuc/RLuc). Luciferase activity readings demonstrated that the wild type IRES vector (pRF-IR) was able to enhance the expression of the downstream located *Firefly *luciferase cistron by ~20 fold as compared to the control vector pRF (Fig. 1C). Deletion of the PPT1 element in the plasmid pRF-IRDel1 reduced luciferase activity by ~2 fold. The deletion of PPT2 sequence in plasmid pRF-IRDel2 alone, or the simultaneous deletion of PPT1, PPT2 and the intervening sequence in pRF-IRDel3 reduced the luciferase activity to background. This effect was further confirmed by Western blotting using deletion constructs. In all constructs, the *Firefly *luciferase gene was replaced by the EGFP coding region. The wild type IRES construct pRE-IR and the deletion constructs, pRE-IRDel1, pRE-IRDel2 and pRE-IRDel3, along with the control vector pRE were transiently transfected into the N2A cells (Fig. 2A). Fourty eight hours post transfection cytosolic cell extracts were prepared and 30 μg of the total protein was separated by 10% SDS-PAGE. Immunodetection with the anti-GFP antibody showed an enhanced expression of EGFP in the construct pRE-IR with the wild type IRES element (Fig. 2B) as compared to the control vector pRE. The deletion of the PPT1 motif induced a moderate decrease in the expression of EGFP as compared to pRE-IR. The deletion of the PPT2 motif alone or a deletion of both PPT1 and PPT2 reduced EGFP expression below the detection limit. This finding confirmed the profound effect of PPT2 on IRES activity consistent with the result obtained by the reporter assay described above.

### Polypyrimidine tract binding protein (PTB) plays an essential role in the IRES activity mediated by PPT1 and PPT2

**Figure 3:**
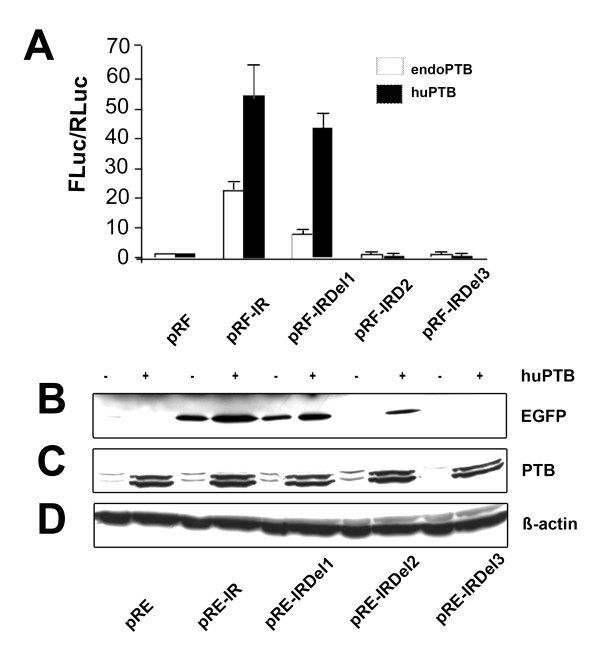
**Overexpression of PTB enhances IRES activity**: A) IRES activity of Di-cis constructs (see Fig. 1B) transiently transfected into N2A cells either in the presence of endogenous PTB or overexpressed human PTB (huPTB) by co-transfection with the pC1-PTB construct. IRES activity is represented as the ratio of *Firefly *to *Rennila *luciferase (FLuc/RLuc), with the activity of control vector pRF set to "1". Each construct was tested 4 times and each experiment was done in triplicates. Data are expressed as mean ± SEM. B) Western blot of Di-cis constructs as summarized in Fig. 2A. All constructs were transiently transfected into N2A cells in the presence of either endogenous PTB (-) or over-expressed huPTB (+). 30 μg of total cytosolic proteins were resolved by 10% SDS-PAGE and immunodetection was done using anti-GFP (1:2,000) as primary antibody and peroxidase labeled anti-mouse IgG (1:7,500) as secondary antibody. C) Western blot of endogenous PTB and over-expressed huPTB from transiently transfected N2A cells. Immunodetection was done by using anti-PTB (1:1,000; Invitrogen) as primary antibody and peroxidase labeled anti-mouse IgG (1:7,500, Jackson ImmunoResearch) as secondary antibody. Note: endogenous and over-expressed huPTB is detected as a doublet band. D) Western blots applying β-actin served as loading control.

The polypyrimidine tract binding protein (PTB) is known to bind to polypyrimidine tracts [34]. We assumed a role of PTB in binding to the two polypyrimidine tracts and thereby modulating IRES activity. This hypothesis was tested by overexpression of the human PTB (huPTB) in N2A cells together with the wild type IRES or PPT deletion constructs (Fig. 3A). After transient transfection into N2A cells, *Rennila *and *Firefly *luciferase activity was measured and ratios calculated. Luciferase readings showed that co-transfection of the vector pC1-PTB encoding the exogenously expressed huPTB with the wild-type IRES construct pRF-IR stimulated luciferase activity ~3 fold as compared to control vector pRF. Similarly, co-transfection of the PPT1 deletion construct, pRF-IRDel1 and pC1-PTB, showed a ~4 fold increase in the luciferase activity. In contrast, simultaneous expression of the PPT2 deletion constructs pRF-IRDel2 and pRF-IRDel3 with huPTB did not affect luciferase activity (Fig. 3A). These results demonstrate that PPT2 is crucial for PTB mediated IRES activity, while PPT1 seems to play a subsidiary role.

A direct correlation between the expression of PTB and the activity of the IRES element was substantiated by Western blots using the EGFP containing Di-cis constructs. The wild type IRES construct pRE-IR and the deletion constructs were transiently co-transfected with the pC1-PTB vector into N2A cells (Fig 3B). Cell extracts were prepared 48 hrs after transfection and 30 μg of the total protein was separated on 10% SDS gel. Immunodetection with anti-GFP and anti-PTB antibodies showed that the huPTB over-expression along with the wild type IRES element resulted in an enhanced expression of EGFP as compared to endogenous expression of PTB. Co-expression of huPTB and the PPT1 deletion construct promoted an increased EGFP expression, which appeared only slightly reduced when compared to the wild type IRES element. The deletion of the PPT2 motif, however, led to a profound reduction of EGFP protein expression. The double mutant lacking PPT1 and PPT2 was indistinguishable from the control vector pRE demonstrating that both PPT motifs are necessary to mediate the full PTB effect, but with different efficiency.

### Polypyrimidine tract I (PPT1) and polypyrimidine tract II (PPT2) are important for the IRES mediated expression of the p11-CT of Cx55.5

**Figure 4:**
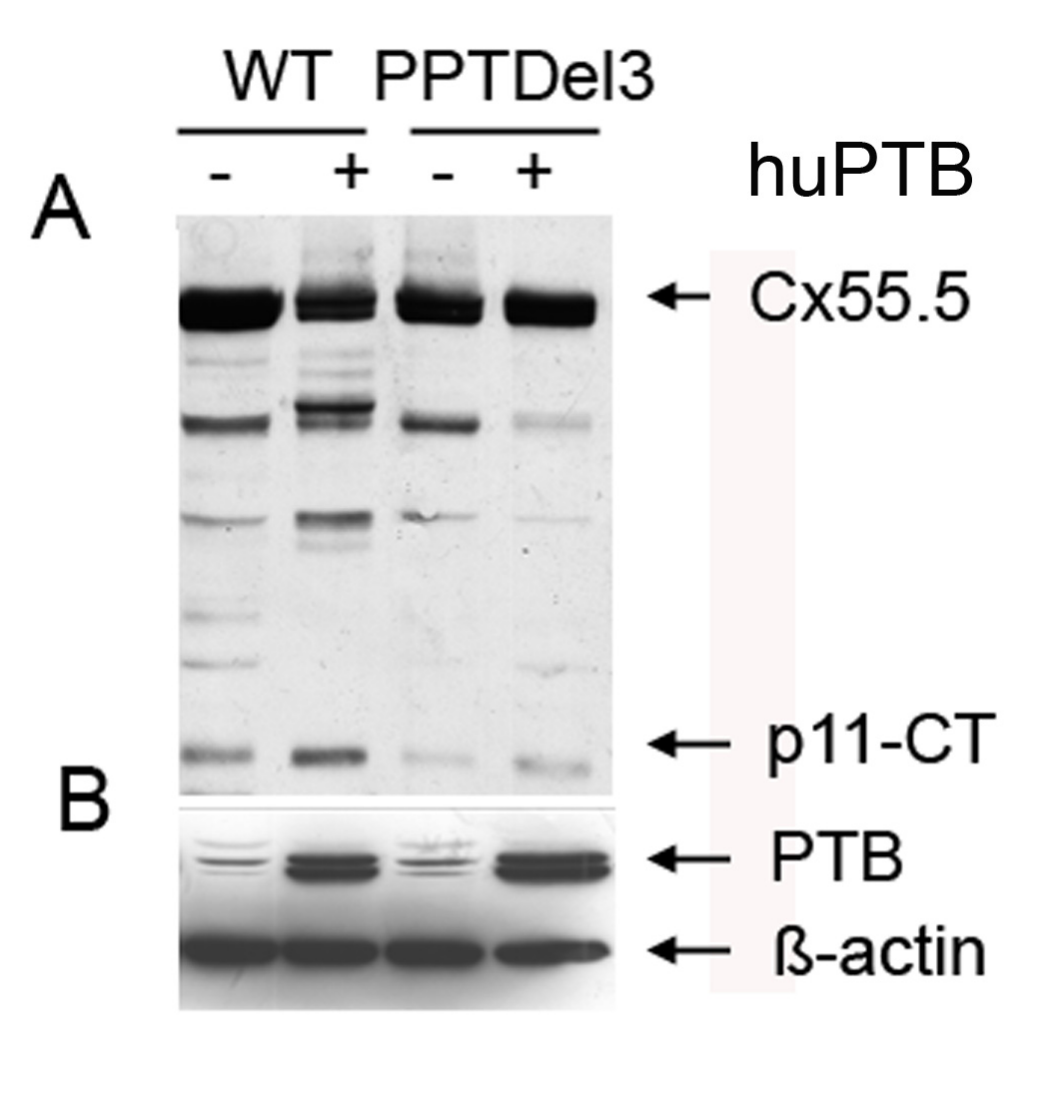
**Polypyrimidine tracts and polypyrimidine tract binding protein (PTB) regulate the expression of p11-CT**. **A) **Western blot of wild type Cx55.5-EGFP (WT) and PPT1 and PPT2 deleted (WT-PPTDel3) fusion constructs transiently transfected in N2A cells in the presence of either endogenous PTB (-) or overexpressed PTB (+). Immunodetection was performed by using anti-GFP antibody as described above. B) Western blot detection of endogenous and huPTB using anti-PTB antibody as described above and with β-actin as loading control.

The importance of PTB and polypyrimidine tracts for the expression of the IRES-dependent translation of the carboxy-terminal fragment (p11-CT) of Cx55.5 was also analyzed using the mutant deficient for both polypyrimidine tract binding sites (PPTDel3). The mutated construct and the wild type Cx55.5-EGFP (WT) construct were transiently transfected into N2A cells either in presence or absence of over-expressed huPTB. 48 hrs post-transfection cytosolic cell extracts were prepared and 30 μg of the total protein was separated on 10% SDS gels. Immunodetection with anti-GFP showed that the deletion of the PPT1 and PPT2 motif reduced the expression of p11-CT as compared to the wild type construct (Fig. 4). Furthermore, over-expression of huPTB with the wild type Cx55.5 construct resulted in the expected enhanced expression of p11-CT, while the over-expression of huPTB with the PPTDel3 mutant promoted p11-CT expression to levels below that of the wild type construct.

### A specific ribonucleic-protein complex (RNP) assembles on the Cx55.5 IRES element

**Figure 5:**
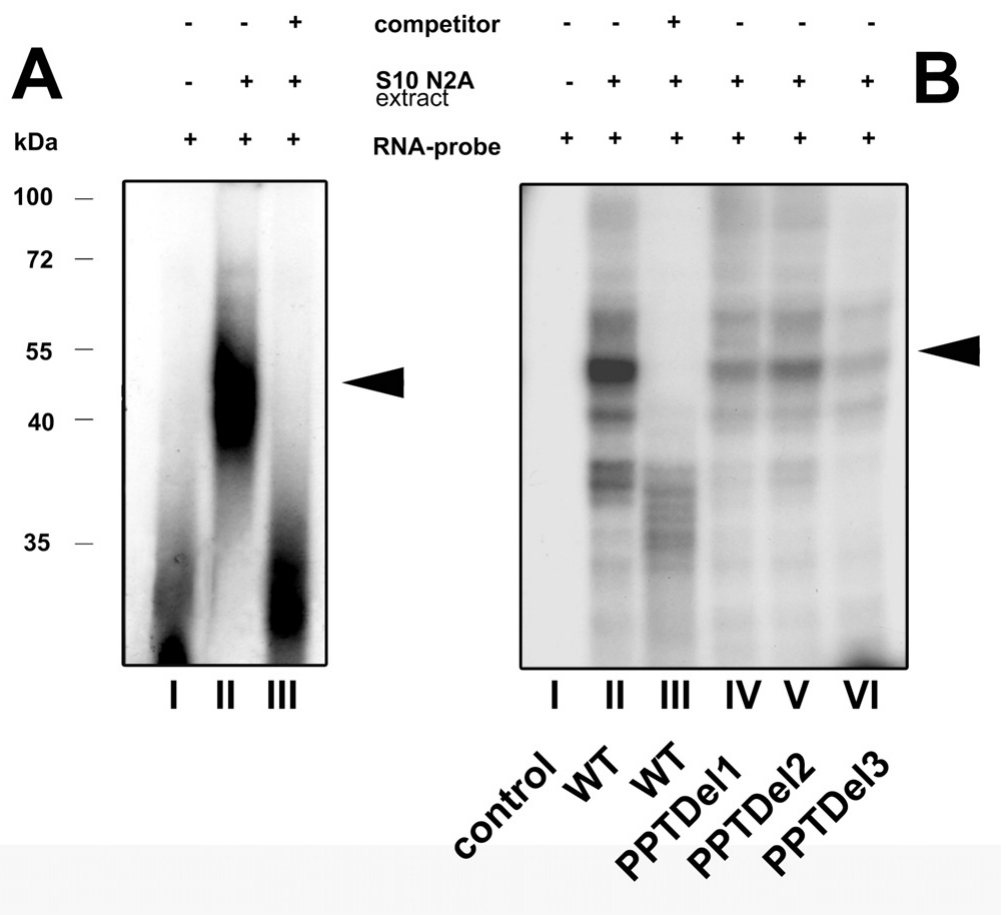
**RNA-EMSA reveals a RNA-protein complex on the IRES element**: A) RNA-EMSA of wild type IRES. Internally labeled ^32^P RNA-probes were incubated with S10 extract from N2A cells. RNA-protein complexes, resolved on a 4% non-denaturing polyacrylamide gel were visualized by autoradiography. The presence of competitor (50 fold molar excess), S10 N2A extract and radiolabeled RNA probe is indicated by (+/-). The position of the RNA-protein binding complex is indicated by the arrow. B) UV cross-linking of RNA probes with S10 N2A extract: RNA-protein complexes were formed as indicated in (A) and samples were subjected to UV cross-linking followed by subsequent RNase treatment and resolved by 10% SDS PAGE. The composition of each sample is indicated as shown in A). Triangles on the right represent specific RNA-protein complexes and the numbers on the left represent a protein molecular weight marker kDa. Densitometrical analysis revealed a reduction to 39% (lane IV), 47% (lane V) and 27% (lane VI) with the wild type condition (lane II) set to 100%.

We used the RNA electromobility shift assay (RNA-EMSA) to determine whether cellular proteins recognize the Cx55.5 IRES element. For this purpose, a radiolabeled RNA probe was incubated with a S10 cytosolic N2A protein extract. As shown in Fig. 5A the cytosolic S10 extract retarded the migration of the RNA probe, leading to the formation of a single dominant RNA-protein complex when compared to the control reaction without protein. Formation of this complex was effectively competed by the inclusion of a 50-fold molar excess of homologous unlabeled competitor RNA.

Additionally, UV cross-linking experiments were performed to further characterize the RNA-protein complexes that assemble at the IRES element (Fig. 5B). When the wild type IRES probe, or its deletion mutants were incubated with the S10 cytosolic N2A protein extract and UV crosslinked several distinct RNA-protein complexes were detected after SDS-PAGE. The crosslinked complexes had apparent molecular masses in the range from 35 to 60 kDa. A prominent band showed an apparent molecular weight of ~55 kDa (Fig. 5B). The formation of the cross-linked RNA-protein complexes was prevented by the inclusion of a 50 fold molar excess of unlabeled homologous competitor RNA. When the deletion mutants PPTDel1, PPTDel2 and PPTDel3 were applied a reduction of the protein complex at around ~55 kDa became apparent, indicating that the deletion of the polypyrimidine tracts weakens the binding of a major RNA binding protein.

### Purified GST-PTB fusion protein is able to bind the IRES element

**Figure 6:**
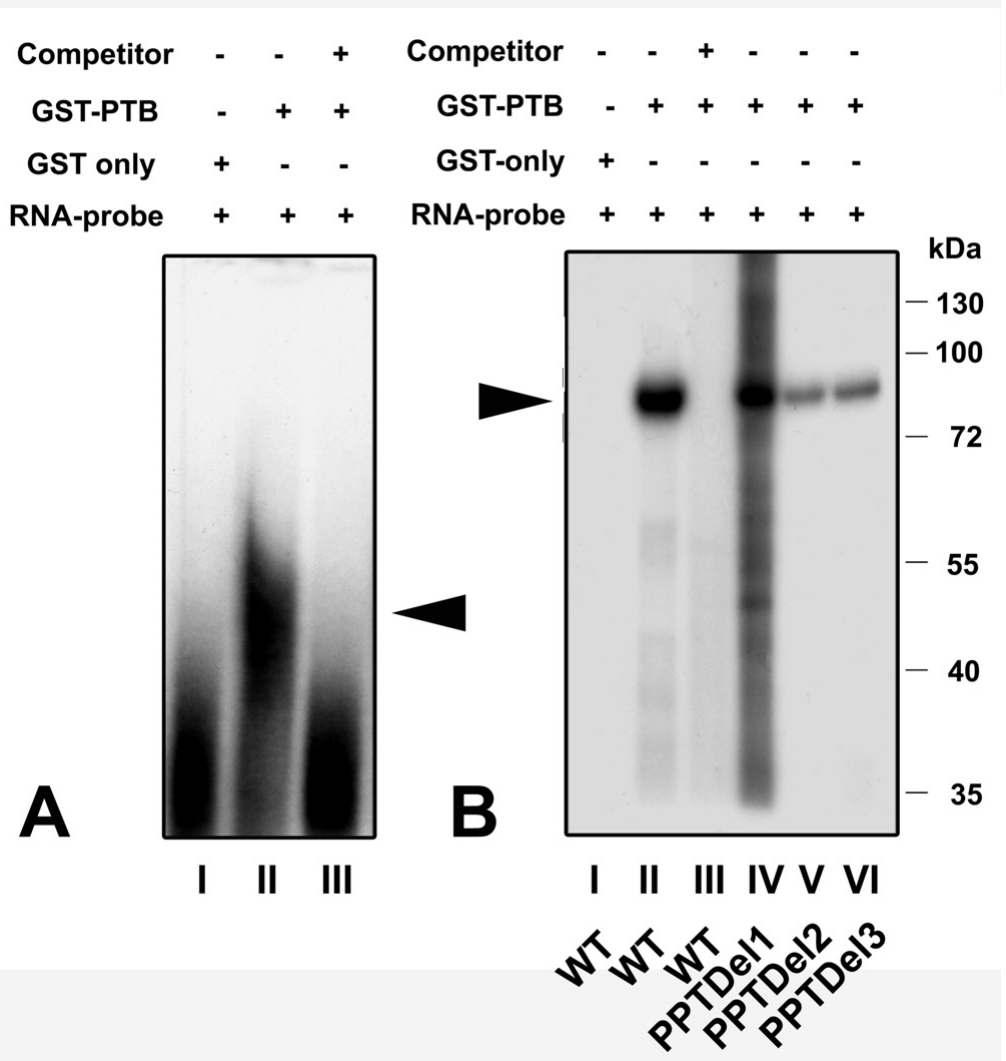
**Recombinant GST-PTB fusion is able to bind the IRES element**: A) RNA-EMSA of wild type ^32^P labeled IRES RNA with: lane I) ~50 μg of purified GST only, lane II) 0.3 μg of purified GST/PTB fusion protein and lane III) cold competition of RNA-protein complex formed in (II) by 20 fold molar excess of unlabeled RNA. The triangle indicates the position of the RNA-protein complex. B) UV cross-linking of GST-PTB to the IRES element. Lane I) GST plus wild type IRES element. Lane II) GST-PTB fusion protein plus wild type IRES element. Lane III) cold competition of (II) using 50 fold molar excess of unlabeled RNA. Lane IV) PPTDel1, lane V) PPTDel2 and lane VI) PPTDel3 IRES mutants. A RNA-protein complex of about 84 kDa was detected in all cases (see triangle), but significantly less in PPTDel2 and PPTDel3 deletion mutants. This complex corresponds to a fusion protein of PTB (57 kDa) and GST (27 kDa). Numbers on right site represent a protein molecular weight marker in kDa.

The prominent RNA-protein complex of ~55 kDa suggested that PTB with an apparent molecular weight of 54 kDa constitutes the protein binding to the IRES element. We evaluated this assumption with a recombinant GST-PTB fusion protein, which was purified to homogeneity and subjected to RNA-EMSA. The wild-type IRES RNA probe and the deletion mutants were incubated with GST alone or with the GST-PTB fusion protein. As shown in (Fig 6A), GST-PTB was able to retard the migration of the RNA probe while GST alone did not show any effect. The formation of the GST-PTB RNA complex was prevented by inclusion of 20 fold molar excess of homologous unlabeled competitor RNA.

The RNA-EMSA data were further confirmed by UV cross-linking of the GST-PTB fusion protein to the wild type IRES RNA, which resulted in the formation of a single RNA-protein complex of ~86 kDa (Fig 6B). This complex was effectively competed by adding 20 fold molar excess of unlabeled homologous RNA. No RNA-protein complex was formed by UV cross-linking GST alone to the IRES element, which excludes that the GST protein itself has RNA binding properties. The PPTDel1 IRES RNA showed a reduced binding when compared to the wild type IRES RNA. This effect was even more pronounced when the PPTDel2 and PPTDel3 mutants were subjected to cross-linking (Fig. 6B).

### PTB interacts with zfCx55.5 mRNA in vivo under physiological conditions

**Figure 7:**
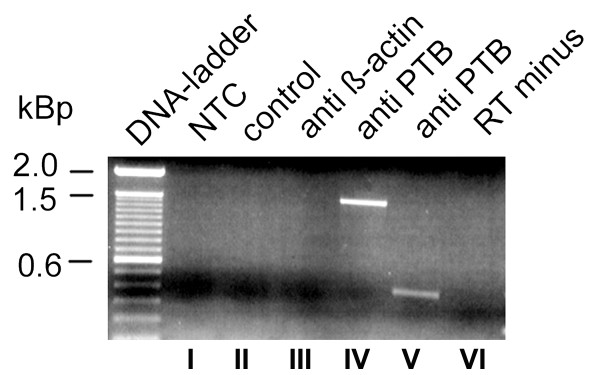
**PTB antibodies precipitate Cx55,5 mRNA from transiently transfected N2A cells**: Binding of PTB to Cx55.5 in living cells was tested 36 hrs post transfection. Crosslinked cell lysates were subjected to immunoprecipitate Cx55.5 RNAs using anti-PTB or anti-β-actin antibodies. RNAs binding to the antibody captured proteins were heat released, reversed transcribed and PCR amplified using primers specific for the Cx55.5 coding region. Lane I) NTC, no template control, lane II) control, PCR of cDNA reactions obtained from the samples immunoprecipitated with Protein A sepharose beads only (without any antibody), III) PCR reaction of cDNA obtained from the RNA immunoprecipitated from anti β-actin coated beads, IV) PCR of cDNA obtained from RNA reverse transcribed with primer pairs specific for the entire coding region (1497). V) cDNA from RNA reverse transcribed with nested primers for the carboxy-terminus fragment (400 bp). VI) RT-minus sample; RNA immunoprecipitated with PTB antibodies without reverse transcription.

To prove that PTB interacts with the Cx55.5 mRNA in living cells under physiological conditions, ribonucleoprotein immunoprecipitation (RNP-IP) assays were performed. N2A cells were transiently transfected with a Cx55.5 expression vector. 36 hrs post transfection cells were harvested in PBS and incubated with formaldehyde to form stable RNA-protein complexes. Cells were lysed and PTB was immunoprecipitated from cell lysates using PTB specific antibodies. The RNA was extracted, reverse transcribed to generate cDNA and subjected to polymerase chain reaction (PCR) with primers specific to the Cx55.5 coding region. The specificity of this approach was proven using β-actin antibody coated beads and a void control with beads only. As shown (Fig. 7), a specific DNA band of about 1497 bp corresponding to the full length Cx55.5 coding region was PCR amplified using the cDNA obtained from the immunoprecipitated RNA. To further confirm the specificity of the immunoprecipated RNA, nested primers specific for the carboxy-terminal domain of Cx55.5 were employed that led to the amplification of the expected carboxy-terminal fragment of ~400 bp (Fig. 7). No amplicons were found in controls using the β-actin loaded and void beads. Thus, the RNP-IP assay provides additional evidence that PTB is able to interact with the Cx55.5 mRNA in living cells under physiological conditions and strengthens our assumption that PTB constitutes a crucial factor involved in activating IRES mediated translation of this neuronal connexin.

## Discussion and conclusion

Internal ribosome entry sites (IRES) are RNA complexes with extensive secondary structures. Several conserved motifs have been described to be essential in the regulation of IRES activity among which polypyrimidine tracts are well documented [36]. Polypyrimidine tracts are involved in the regulation of mRNA translational mechanisms and the importance of oligopyrimidine sequences have already been reported upstream of AUG initiation codons in picornaviruses [37,38] and in the hepatitis C virus [39-41]. In continuation of previous studies, we analyzed the internal IRES element of Cx55.5 for sequence motifs of trans-acting factor(s), which are important for the function of IRES activities. Sequence analyses showed the presence of two stretches of polypyrimidine tracts, PPT1 and PPT2 flanking the in frame IRES sequence of Cx55.5. To investigate the influence of these polypyrimidine tracts on the activity of the IRES element, we deleted PPT1 and PPT2 either alone or in combination. Subsequent reporter assays and Western blot analyses showed that deletion of PPT1 had an appreciable effect on the IRES activity, while deletion of PPT2 results in complete loss of IRES activity. This result indicates that the 14 bp stretch of PPT2 serves an important function in determining IRES activity of Cx55.5 albeit by altering the RNA fold.

In addition to their requirements for eukaryotic initiation factors, the efficiency of most IRES elements is augmented by non-canonical initiation factors know as ITAFs (internal initiation trans-acting factors) [42,43]. Since our initial results indicated an involvement of polypyrimidine tracts in defining the IRES activity of Cx55.5, we investigated the role of the polypyrimidine tract binding (PTB) protein. The IRES activity of the wild type IRES element and its deletion mutants was analyzed in the presence of endogenous levels of PTB or upon overexpression of the protein by transfection with huPTB. Overexpression resulted in a significant increase of IRES activity of the wild type IRES element. Deletion of the PPT1 element led to a significant reduction as compared to the wildtype, but the IRES element was still responsive in particular when exogenous huPTB was added. Deletion of PPT2 alone or in combination with PPT1 revealed a complete loss of responsiveness. Our results suggest that the polypyrimidine tract 2 (PPT2) is crucial for the IRES activity and that PTB conveys a role in the regulation of the IRES activity of Cx55.5.

A plausible explanation for the critical role of PPT2 in the IRES activity is that it binds to additional crucial trans-acting factor(s), which are important for the recruitment of the ribosomal translational machinery. Alternatively, this tract is important for the specific RNA secondary structure and the role of PTB seems to stabilize the active confirmation of the IRES element through binding to the RNA scaffold [44]. Interestingly, the secondary structure prediction using the RNA folding algorithm mFold [45] of the wild type IRES element and its deletion mutants (see Additional file 1) revealed that the wild type IRES of Cx55.5 has an extended stem-loop structure with semi-conserved Y-like configuration, described also for IRES elements found in some picornaviruses [38,46]. Deletion mutation of PPT1 showed overall a similar structure when compared to the wild type RNA fold with a minor loss of the small stem loop. In contrast, the deletion mutant PPT2 showed a complete remodeling of the structure, which was predicted to be energetically less stable as compared to the wild type IRES RNA fold.

The PTB protein is very well known in regulating IRES activity of both viral IRES and cellular IRES elements [37,38,47,48]. These data are of particular interest keeping in mind that PTB is primarily a nuclear protein, where it plays a role in regulation of splicing events of eukaryotic mRNAs [27]. To become functional in IRES related activities, PTB needs to shuttle from the cell nucleus to the cytoplasm. Recent evidence on the involvement of IRES elements in internal translation of biologically active protein domains makes it essential that this processes need to be regulated [48,49].

Shuttling events of PTB from the cell nucleus to the cytoplasm can be one step where regulation by internal and external factors becomes effective. One such mechanism has been described recently [49]. In this study, protein kinase A phosphorylation (PKA) was found to modulate shuttling of PTB by phosphorylation of a particular serine residue. This modification results in an increase of the cytoplasm-directed transport of PTB from the nucleus and couples the PKA pathway with translocation events of PTB.

In the context with our recent findings, which demonstrated expression of an IRES driven carboxy-terminal fragment (p11CT) of Cx55.5 and its translocation to the nucleus of horizontal cells a link between PTB shuttling and regulated expression of this fragment is challenging. [18,19]. Due to the prominent role of gap junction coupling in horizontal cells in primary processing of visual stimuli and its dependence on dopamine regulation [49-52], it will be of considerable interest to examine whether a cAMP dependent mechanism involving dopamine affects PTB shuttling and contributes to the strength of intercellular horizontal cell coupling. Alternatively, retinoic acid, which exerts a profound effect on synaptic plasticity of horizontal cells and involves PKA phosphorylation to execute its signal transmission potency [53] may comprise another candidate, which could operate through the indicated mechanism.

Aside of the identification of PTB as a protein binding to the Cx55.5 IRES element further binding proteins covering the range of ~35 to 60 kDa were identified by UV crosslinking experiments. Some of them are in the size range of proteins, which have been described to bind to other IRES elements [54]. It remains to be established whether these proteins constitute further component of this complex, and whether they are also involved in functional regulation of the Cx55.5 IRES element.

In summary, we demonstrate that the activity of the internal IRES element of the horizontal cell specific gap junction protein Cx55.5 is modulated by two polypyrimidine tracts. We further provide evidence that the polypyrimidine binding protein (PTB) works as a non-canonical factor in the regulation of the IRES activity. The physiological meaning of the Cx55.5 IRES activity and in consequence the generation of the p11-CT protein in horizontal cell function awaits further clarification.

## Methods

### Plasmid construction

The zebrafish Cx55.5 IRES element (631 nt to 989 nt; position relative to the start codon) was cloned into the Di-cistronic vector pRF-IR [19] and mutated using the Transformer Site Directed Mutagenesis Kit (Clontech East Meadow Circle, Palo Alto, CA, USA). A deletion of 9 bp (nt 909 to nt 917) of the polypyrimidine tract 1 (PPT1) was performed with the primer 5'-CCT GAT GCC TAG ATT AAC CCA TCC-3' using plasmid pRF-IR as template. The new construct was designated as pRF-IRDel1. 14 bps of the second polypyrimidine tract (PPT2; nt 928 to nt 941) were deleted by introducing unique Sma I and PvuII restriction sites at 5'- and 3' end of PPT2. The Sma I and Pvu II restriction sites were used to remove the PPT2 sequence in plasmid pRF-IRDel2. For the simultaneous deletion of PPT1, PPT2 and the intervening sequence of 11 bp, a unique EcoRV site was created at the immediate 5'-end of PPT1. The final construct pRF-IRDel3 was obtained after restriction digest with EcoRV and Pvu II followed by religation of the vector DNA. A cartoon of all plasmids generated is shown in Fig. 1A and the Additional File 2. A second set of constructs was generated by transfer of the wild type IRES element (IR) and the three PPT deletion mutants into the intercistronic region of the plasmid pRE [19]. In this vector, the Firefly luciferase was replaced by EGFP as second cistron (see Fig. 2A, Additional file 2).

The vector encoding the GST-human polypyrimidine tract binding protein [pGEX2TK (huPTB)] was a kind gift from Dr. M. Garcia-Blanco (Durham, N.C, USA). The entire human PTB coding DNA sequence (nt 1- nt 1594) was isolated using the EcoRI restriction sites and ligated into the EcoRI site of the pEGFP-C1 vector (BD Biosciences Clontech, CA, USA) to generate the construct pEGFP-C1-PTB. This construct was further manipulated and the EGFP gene at the N-terminus of the PTB gene removed. The construct lacking EGFP was termed pC1-PTB. The wild type Cx55.5-EGFP (WT) fusion construct in pEGFP-N3 vector [19] was manipulated to delete PPT1 and PPT2 simultaneously as described above while preserving the reading frame of the mutated protein. The mutant construct was named PPTDel3 (see Additional File 2).

Monocistronic vector constructs lacking the *Renilla *luciferase (RLuc) were generated for in vitro transcription using the T7 promoter. For this purpose, the RLuc gene was released from the parental vectors including the IRES sequence or the three deletion mutants by restriction digest with Nhe I and EcoRI. The plasmid backbones lacking the RLuc gene were isolated and religated after Klenow fragment treatment. The resulting plasmids were pT7-IR, pT7-IRDel1, pT7-IRDel2 and pT7-IRDel3 (see Additional File 2).

### Cell culture, transfection, and reporter-assays

Neuro2A cells purchased from the ATCC collection (Manassas, VA, USA) were grown in 78 cm^2 ^tissue culture dishes (Becton Dickinson, Heidelberg, Germany). For determination of IRES activity, 2×10^4 ^N2A cells were plated in 96 well flat bottom plates (Becton Dickinson) and processed as previously described [19].

### Western blot analysis

For western blot analysis, 2 × 10^5 ^N2A cells were seeded in 12 well plates (Becton Dickinson). After twelve hours, transient transfections were performed using a 300 ng plasmid DNA and the Effectene^® ^transfection protocol (Qiagen). For co-transfection, 100 ng of pC1-PTB plasmid was included in the transfection mixture when indicated. Further steps were performed as described by Ul-Hussain et al. [19].

### Protein expression and purification

The expression vector constructs pGEX2TK(huPTB) and the parental control plasmid pGEX6P2 were transformed into the BL21 host strain (Stratagene). Fusion protein expression was induced for 16 hrs at 30°C after induction with 1 mM IPTG. Bacteria were collected at 5,000 g and protein lysates prepared using the French Press 2-FA-031 (Thermo Spectronic, Rochester, NY, USA). Precleared lysates were subjected to affinity chromatography using the ÄKTA-LC System, GST-Trap FF columns and standard conditions as recommended by the manufacturer (Amersham Biosciences). Peak fractions were desalted using HITrap desalting columns (Amersham Biosciences), concentrated using Amicon Ultra-4 columns (Millipore), and protein purity assessed by conventional SDS-PAGE.

### In vitro transcription

For in-vitro transcription, pT7-IR, pT7-IR 1, pT7-IR 2 and pT7-IR 3 were linearized 3' to the IRES element using the Xho I restriction site. For labeling RNA, in vitro transcription was performed using the MAXIscript T7 Kit (Ambion, Inc., Austin TX, USA) in accordance to the manufactures instructions. 5 μl of ^32^P CTP (10 μCi/μl; 3000 Ci/nmole) (Amersham) was included in the reaction mixture. Unlabeled competitor RNA was synthesized using MEGAscript™ T7 Kit (Ambion). Labeled RNA probes were purified using Sephadex G50 columns (Amersham).

### RNA-EMSA

Internally labeled wild type IRES RNA and the various deletion mutants were used for the electromobility shift assay (EMSA). Approximately 20,000 cpm of labeled RNA probes were mixed with 30 μg of cytosolic protein prepared from N2A cells or 0.3 μg of purified GST-PTB fusion protein and further steps were performed as described in [55]. After electrophoresis the gel was transferred to Watmann paper, dried and visualized by autoradiography.

### UV-cross linking

The RNA-protein complexes for UV-cross linking were prepared as described above. For cold competition, unlabeled RNA was added 5 minutes after the addition of the RNA probe. After 30 minutes at RT the samples were transferred to ELISA plates and irradiated for 30 minutes at 4°C with UV light (312 nm) in an UV Stratalinker model 1800 (Stratagene). 2 μl of RNase A (10 mg/ml), and 1 μl of RNase T1 (100 units) (Fermantas), was added and samples incubated at 37°C for 30 minutes. RNA-protein complexes were resolved by 10% SDS PAGE for 3 hours at 200 V. Subsequently, the gel was dried under vacuum and visualized by autoradiography. Densitometrical analysis was performed using ImageJ software.

### Ribonucleoprotein Immunoprecipitation Assay (RNP-IP)

N2A cells (2 × 10^6^) in 100 mm dishes were transiently transfected with the expression vector having Cx55.5 cDNA. 36 hours after transfection, cells were harvested in PBS and washed twice with 5 ml of PBS, resuspended in 5 ml of phosphate-buffered saline and processed as described (53). In brief, Protein A Spharose beads were coated either with ant-PTB or anti-β-actin antibody for 2 h at 4°C followed by extensive washing with RIPA buffer. Bound RNA was released from crosslinked protein-RNA complexes by heating the samples at 70°C for 45 min followed by RNA extraction using Trizol reagent according to the manufactures protocol (Invitrogen). The RNA was reverse transcribed with random hexamers and MMLV reverse transcriptase (Invitrogen) according to the manufacturer's protocol. PCR was performed using two sets of primers specific to the coding region of Cx55.5. Set I: sense 5'-ATG GGA GAC TGG AAC TTT CTT GG-3' and anti sense 5'-AAT TTG TAA GTC TGT GGG AGC-3'. The amplicon size is 1497 bp. Set II: sense 5'-CAA TGC ATA GCT GGC ATT TCA TTG-3' and antisense 5'-GTG GAG TGA CAG AGT TGC AAG-3'. The amplicon generated by Set II corresponds to 400 bp of the carboxy-terminal domain of Cx55.5.

## Authors' contributions

MUH conceived the study, carried out the molecular genetic studies and drafted the manuscript. GZ: participated in the molecular genetic studies and helped to draft the manuscript. RD: participated in its design and coordination and helped to finalize the manuscript. All authors read and approved the final manuscript.

## Supplementary Material

Additional file 1 **Cx55.5 RNA fold prediction models indicating the impact of polypyrimidine tracts on the RNA folding**. Secondary structure prediction using mFold algorithm of the wild type IRES element (wt) and the PPT deletion mutants dPPT1, dPPT2, dPPT3 corresponding to IRDel1, IRDel2 and IRDel3 (see Figures 1, 2). Click here for file

Additional file 2 **Summary of plasmid constructs**. Summary table including all plasmid constructs used in the present study. Click here for file
