# Supplementary figures and images for: Characterization of the internal IRES element of the zebrafish connexin55.5 reveals functional implication of the polypyrimidine tract binding protein
Source: BMC Mol Biol. 2008 Oct 23;9:92. doi: 10.1186/1471-2199-9-92 (PMC2579433; doi:10.1186/1471-2199-9-92)

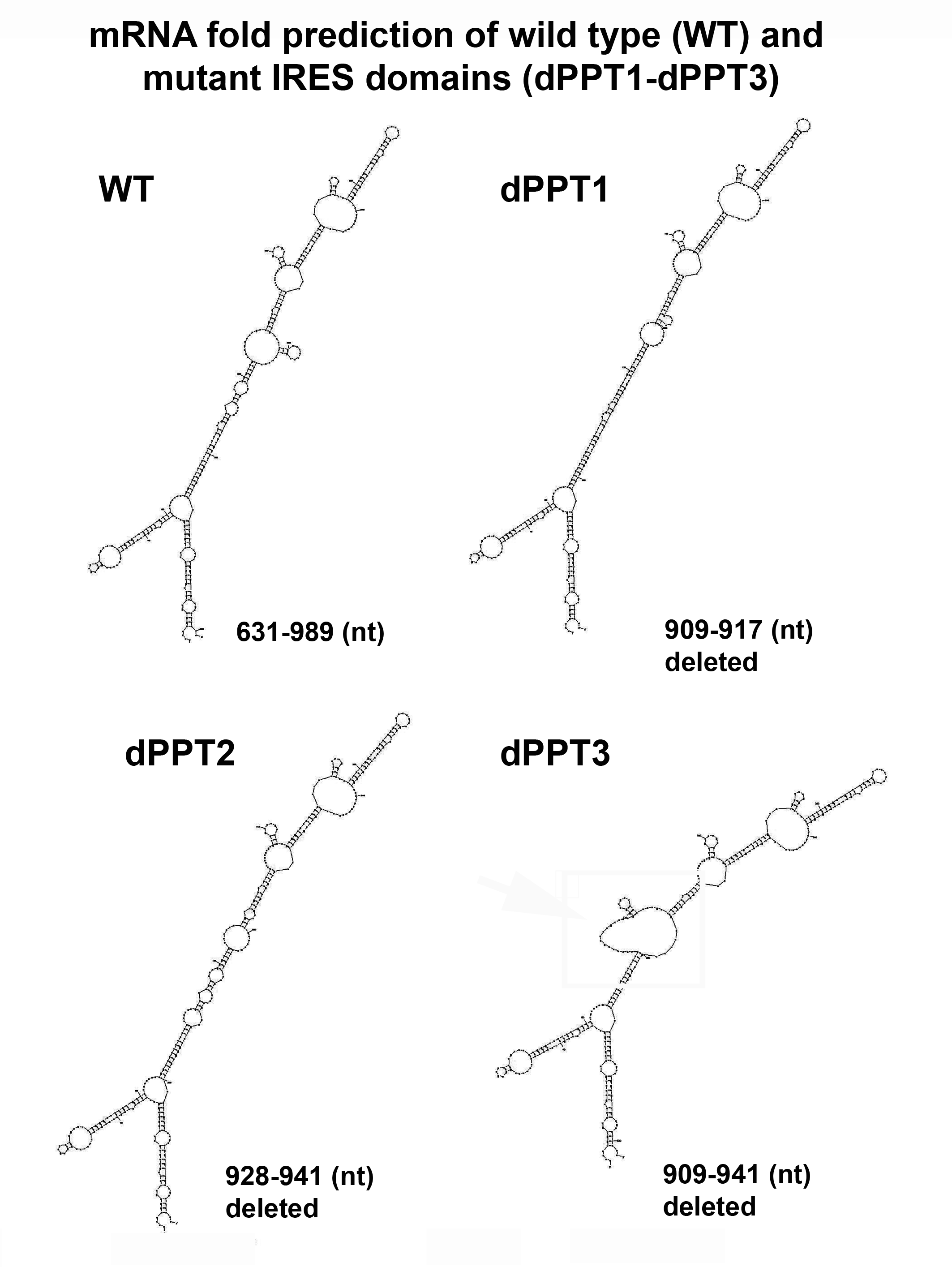

Supplement: Additional file 1 — Cx55.5 RNA fold prediction models indicating the impact of polypyrimidine tracts on the RNA folding. Secondary structure prediction using mFold algorithm of the wild type IRES element (wt) and the PPT deletion mutants dPPT1, dPPT2, dPPT3 corresponding to IRDel1, IRDel2 and IRDel3 (see Figures 1, 2). [file 1471-2199-9-92-S1.tiff]
