# Supplementary material for: Characterization of the internal IRES element of the zebrafish connexin55.5 reveals functional implication of the polypyrimidine tract binding protein
Source: BMC Mol Biol. 2008 Oct 23;9:92. doi: 10.1186/1471-2199-9-92 (PMC2579433; doi:10.1186/1471-2199-9-92)
Supplement: Additional file 2 — Summary of plasmid constructs. Summary table including all plasmid constructs used in the present study. [file 1471-2199-9-92-S2.doc]

**Additional File II:** Summary of plasmid constructs

| name | basic plasmid | Cx55.5 insert (nt) | modification | comment |
| --- | --- | --- | --- | --- |
| pRF-IR | pRF-Di cis | 631-989 | none | wild type |
| pRF-IRDel1 | pRF-Di cis | 631-989 | 909-917deleted | PPT1 deletion |
| pRF-IRDel2 | pRF-Di cis | 631-989 | 928-941 deleted | PPT2 deletion |
| pRF-IRDel3 | pRF-Di cis | 631-989 | 909-941 deleted | PPT1+2 and intervening seq. deleted |
| pRE-IR | pRF-Di cis | 631-989 | none | Firefly gene replaced by EGFP |
| pRE-IRDel1 | pRF-Di cis | 631-989 | 909-917deleted | PPT1 deletion |
| pRE-IRDel2 | pRF-Di cis | 631-989 | 928-941 deleted | PPT2 deletion |
| pRE-IRDel3 | pRF-Di cis | 631-989 | 909-941 deleted | PPT1+2 and intervening seq. deleted |
| pT7-IR | pRF-Di cis | 631-989 | Renilla luciferase cistron deleted | Wild type under the control of T7 promoter |
| pT7-IRDel1 | pRF-Di cis | 631-989 | 909-917deleted | PPT1 deletion |
| pT7-IRDel2 | pRF-Di cis | 631-989 | 928-941 deleted | PPT2 deletion |
| pT7-IRDelΔ3 | pRF-Di cis | 631-989 | 909-941 deleted | PPT1+2 and intervening seq. deleted |
| pWT | pEGFP-N3 | 1-1497 | none | Wild type zfCx55.5-EGFP fusion construct |
| pWT-PPT Del3 | pEGFP-N3 | 1-1497 | 909-941 deleted | PPT1+2 and intervening seq. deleted |
| pC1-PTB | pEGFP-C1 | none | none | cDNA of polypyrimidine tract binding protein (PTB) |
| pGEX2TK(hu PTB) | pGEX2TH | none | none | GST-PTB fusion protein |

- pEGFP-N3 was obtained from BD Biosciences Clontech, CA, USA
- pRF-Di cis was a gift of Dr. Rudolf Werner (Department of Biochemistry and Molecular Biology, University of Miami, School of Medicine).
- pGEX2TK(hu PTB) was a gift of Dr M. Garcia-Blanco (Durham, N.C, USA)
